# Fulminant Guillain–Barré Syndrome and Spontaneous Intraventricular Hemorrhage: A Case Report and Literature Review

**DOI:** 10.3389/fnins.2020.00633

**Published:** 2020-06-30

**Authors:** Jun Hu, Xiaoqian Luo, Yu Wang, Eric Prado, Qinghui Fu, Anwen Shao

**Affiliations:** ^1^Department of Surgical Intensive Care Unit, The Second Affiliated Hospital, School of Medicine, Zhejiang University, Hangzhou, China; ^2^Department of Pediatrics, Children’s Hospital of Zhejiang University School of Medicine, Hangzhou, China; ^3^Loma Linda University School of Medicine, Loma Linda, CA, United States; ^4^Department of Intensive Care Unit, The First Affiliated Hospital, School of Medicine, Zhejiang University, Hangzhou, China; ^5^Department of Neurosurgery, The Second Affiliated Hospital, School of Medicine, Zhejiang University, Hangzhou, China

**Keywords:** Guillain–Barré syndrome, spontaneous intraventricular hemorrhage, etiology, prognosis, review

## Abstract

Guillain–Barré syndrome (GBS) is an acute, immune-mediated inflammatory peripheral polyneuropathy that is characterized by flaccid paralysis. A few cases have reported that GBS can be caused by head trauma or neurosurgery, but it has never been associated with intraventricular hemorrhage. Here, we report an uncommon case of fulminant GBS that occurred after spontaneous intraventricular hemorrhage. A 73-year-old woman was admitted to the hospital after sudden unconsciousness and vomiting. A head computed tomography (CT) scan following the incident showed a newly developed intraventricular hemorrhage, which led to an immediate ventriculostomy. After 5 days, the endotracheal tube was removed. Two days later, the external ventricular drainage tube was also removed. At this time, the patient was alert and the neurological examination was normal. However, the patient suddenly presented with acute respiratory failure and bilateral limb weakness 3 days later. An analysis of the patient’s cerebrospinal fluid (CSF) revealed that albuminocytologic dissociation was present. The patient was treated with intravenous immunoglobulin (0.4 g/kg/day) for 5 days. Despite timely medical intervention in the hospital, the patient passed away 2 months later. After a cerebral hemorrhagic injury, limb and respiratory muscle weakness can occur on occasion in the ICU. In this context, the potential involvement of GBS should not be ignored. Importantly, the pathogenic mechanism of GBS has been discussed for over a century, and it still remains a mystery. We speculate that the TLR4/NF-κB signaling pathway may be involved in the pathogenesis of GBS following intraventricular hemorrhage. The prognosis of most patients with GBS is usually good, but cerebral hemorrhage and mechanical ventilation may serve as risk factors that exacerbate the condition. This case is reported to remind clinicians to consider the possibility of GBS when patients present limb and respiratory muscle weakness after intraventricular hemorrhage, and to provide a starting point to discuss potential mechanisms of GBS after intraventricular hemorrhage.

## Introduction

Guillain–Barré syndrome (GBS) is an acute, immune-mediated inflammatory peripheral polyneuropathy that is characterized by flaccid paralysis. The incidence rates of GBS are 0.8–1.9 cases per 100,000 people per year (Willison et al., 2016). The classic clinical manifestation of this disease is characterized by rapidly evolving, bilateral limb weakness and albuminocytologic dissociation. In addition, about 20–30% of cases may present with respiratory failure, which requires months of intensive care that could potentially aggravate the condition (Goodfellow and Willison, 2016; Willison et al., 2016). Intravenous immunoglobulin and plasma exchange are routinely used to efficiently treat GBS, which results in a good prognosis for most patients with GBS. However, the outcomes of patient are various. Some patients have not fully recovered and lose their quality of life. Nearly 15% of patients are disabled and approximately 4–5% of patients die from complications of this disease (Creange, 2016; Goodfellow and Willison, 2016).

Recently, reports that GBS can be caused by head trauma or neurosurgery have increased, but GBS had never been associated with intraventricular hemorrhage. Herein, we report an uncommon case of fulminant GBS that occurred after spontaneous intraventricular hemorrhage (SIVH). In this case, a 73-year-old woman underwent surgery for SIVH. Several days after her endotracheal tube was removed, the patient presented with acute respiratory failure and bilateral limb weakness. After numerous diseases were excluded, the diagnosis of GBS was made even though it is rarely reported in similar situations. In cases like this, it is difficult to identify the causes of respiratory failure and bilateral limb weakness; the mechanism is still unknown and requires further discussion. Therefore, we report this case to remind clinicians to pay more attention to the mechanism, diagnosis, differential diagnosis, therapy, and prognosis of similar cases. We also provide a starting point to discuss potential mechanisms that lead to GBS after intraventricular hemorrhage. However, additional evidence is needed from similar cases for the elucidation of these potential mechanisms.

## Case Report

**FIGURE 1:**
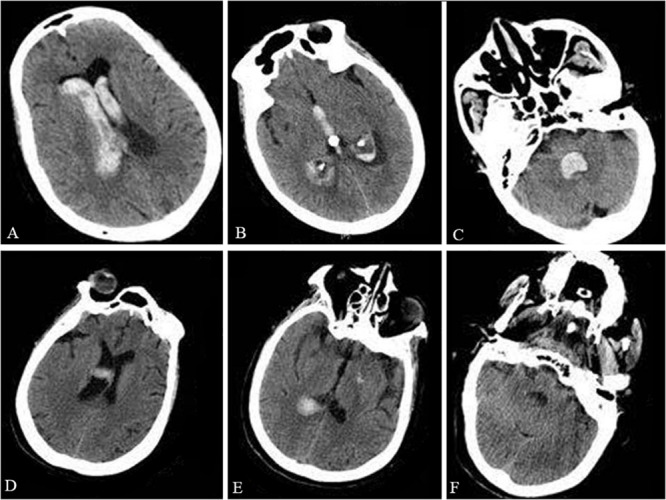
Preoperative and postoperative CT. The preoperative head CT showed intraventricular hemorrhage in the lateral ventricles **(A)**, third ventricle **(B)**, and fourth ventricle **(C)**. The postoperative head CT taken during the patient’s acute respiratory failure and shortness of breath excluded an intracranial re-hemorrhage in the lateral ventricles **(D)**, third ventricle **(E)**, and fourth ventricle **(F)**.

A 73-year-old woman was admitted to the hospital after sudden unconsciousness and vomiting without a preceding trauma. The patient was able to open her eyes and bend her limbs, but only after painful stimuli. The patient’s Glasgow coma score was 8 and a head computed tomography (CT) scan showed intraventricular hemorrhage (Figures 1A–C). An external ventricular drain was placed immediately and then the patient was transferred to the Intensive Care Unit (ICU). Five days later, the patient regained consciousness and became cooperative. At this time, the neurological examination failed to show any strength deficits. The endotracheal tube was removed and the patient was transferred to the general ward for enhanced recovery. Two days later, a head CT scan revealed the elimination of the intraventricular hemorrhage, resulting in the external ventricular drainage tube being removed.

However, 3 days later, the patient presented acute respiratory failure with shortness of breath. The patient’s oxygen saturation was approximately 75% and her temperature was 38.5°C. In addition, the patient’s blood pressure was 99/60 mmHg with a heart rate of 110 bpm. Arterial blood gas analysis showed that the PO_2_ was 59 mmHg and the PCO_2_ was 49 mmHg. An emergent head CT scan excluded intracranial re-hemorrhage (Figures 1D–F). A pulmonary artery embolism was also excluded by pulmonary CT angiography (CTA). Despite an attempt of non-invasive ventilation, the patient deteriorated and required intubation with mechanical ventilation. During the following days, the patient was conscious, but with a Glasgow coma score of 4 + T + 6 and bilateral limb weakness (muscle strength was 1/5 the strength in the upper and lower extremities). Tendon reflexes were absent. The neck muscle strength and sensation in the limbs were normal. Eye movements and corneal reflexes were intact. Serum sodium was within normal limits, but the serum potassium was low (2.65 mmol/L). After the correction of hypokalemia, the patient’s bilateral limb strength worsened (muscle strength was 0/5, the strength in the upper and lower extremities). A lumbar puncture was then performed because of the worsening clinical condition. Examination of the cerebrospinal fluid (CSF) showed albuminocytologic dissociation with a cell count of 8 × 10^6^ cells/L (normal range is below 8 × 10^6^ cells/L) and a total protein level of 107.3 mg/dl (normal range is 8–43 mg/dl). An extensive series of serum antibodies against ganglioside were negative, including GQ1b, GT1b, GD1b, GD1a, GM1, GM2, and GM3. The result of a creatinine kinase test was within normal limits (48 U/L; normal range is below 145 U/L). Unfortunately, the electromyogram failed because of substantial logistical difficulties. In addition, the relatives of the patient denied any history of recent viral illnesses and any other previous diseases.

**FIGURE 2:**
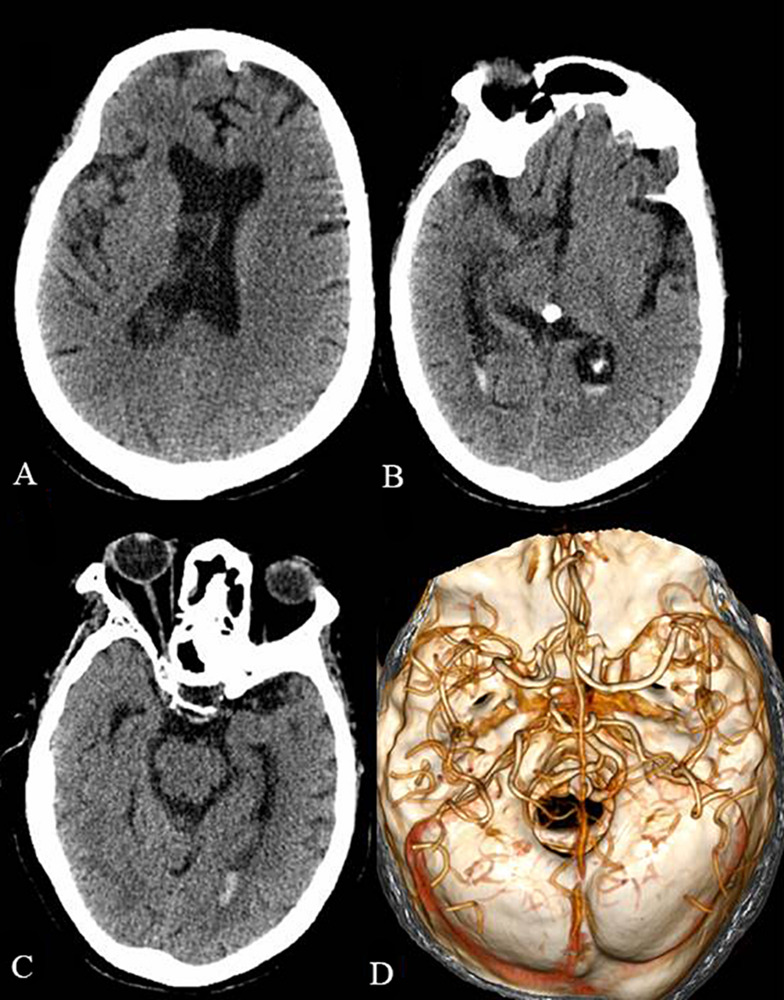
The head CT and CTA 1 month after the operation of the intraventricular hemorrhage. The intraventricular hemorrhage was significantly decreased in the lateral ventricles **(A)**, third ventricle **(B)**, and fourth ventricle **(C)**. The head CTA showed cerebral arteriosclerosis and excluded arterial aneurysm and cerebrovascular malformation **(D)**.

The patient was treated with intravenous immunoglobulin (0.4 g/kg/day) for 5 days. Despite therapeutic intervention, the limb weakness persisted and the patient required continuous ventilator assistance beyond the 5 days of immunoglobulin treatment. Subsequently, a tracheotomy was performed. One month after the operation for the intraventricular hemorrhage, a head CT scan revealed a significant decrease of the intraventricular hemorrhage (Figures 2A–C). A head CTA, which was performed to identify the cause of the cerebral hemorrhage, only showed cerebral arteriosclerosis (Figure 2D). Five days later, the patient was transferred to the local hospital for further rehabilitation therapy. Unfortunately, the patient passed away after the 1 month follow-up.

## Discussion

GBS was first reported by Guillain, Barré, and Strohl in 1916 (Goodfellow and Willison, 2016). GBS has several subtypes including acute inflammatory demyelinative polyradiculoneuropathy (AIDP), acute motor axonal neuropathy (AMAN), acute motor-sensory axonal neuropathy (AMSAN), and Miller Fisher syndrome (MFS). AIDP is the most common type in GBS; AMAN usually presents pure motor nerve damage, especially motor axons; AMSAN mainly presents sensory and motor axonal damage; unlike the classic GBS, MFS is characterized by ophthalmoplegia, ataxia, and disappearance of tendon reflexes. The pathogenic mechanism has been discussed for more than a century. The disease can be induced by a number of microorganisms, including *Campylobacter jejuni*, cytomegalovirus, leptospirosis, Epstein–Barr virus, Hepatitis E virus, and Zika virus (Creange, 2016; Willison et al., 2016; Kobawaka Gamage and Fernando, 2018). Molecular mimicry of gangliosides caused by these pathogenic microorganisms may take part in the occurrence and development of GBS (Goodfellow and Willison, 2016; Willison et al., 2016). Recent research has considered that anti-ganglioside antibodies may be associated with GBS. The complement activated by these antibodies leads to the formation of the membrane attack complex and disruption of nodal architecture at the node of Ranvier as well as neuronal and glial injury at the neuromuscular junction (Goodfellow and Willison, 2016). The relationship between anti-ganglioside antibodies and AMAN may be present, but the pathophysiology is still not clear for the other subtypes. Importantly, the exact mechanisms of GBS are still mysterious, demonstrating the need for further research and case reports.

In our case, the patient presented with GBS after spontaneous intraventricular hemorrhage. There are also a few cases that have reported that GBS can be caused by head trauma, neurosurgery, or other cerebral hemorrhagic injury (Rivas et al., 2008; Yardimci et al., 2009; Tan et al., 2010; Song et al., 2012; Mantero et al., 2013; Zhang and Li, 2014; Wu et al., 2016; Li et al., 2017). Table 1 reviews the GBS cases that have developed after cerebral hemorrhagic injury (CHI). Moreover, there are some GBS cases that have been described before CHI (Table 2; Gande et al., 1999; Doss-Esper et al., 2005; Chang et al., 2014; Wei et al., 2015). Some of the possible mechanisms of GBS after CHI are the acute inflammation triggered by hemoglobin infiltration, disturbance of cellular humoral immunity after brain injury, and the stress state after hemorrhage (Wu et al., 2016). However, the molecular mechanism leading to GBS is still unclear.

**TABLE 1:** GBS after cerebral hemorrhagic injury (CHI).

**References**	**Sex/age**	**Types of CHI**	**Subtype of GBS**	**Vascular dysfunction**	**Treatments**	**Outcome**	**Follow-up time**	**Presentation of prognosis.**
Rivas et al., 2008	M/55	Subdural hemorrhage	AIDP	Unknown	PE; MV	Partial	6 month	Distal extremity contractures and paresthesias
Yardimci et al., 2009	F/75	Subdural hemorrhage	AMSAN	None	PE	Completed	4 month	–
Tan et al., 2010	M/44	SAH	AMAN	Unknown	IVIG	Partial	10 month	Weakness of distal bilateral limb
Song et al., 2012	F/52	Pontine hemorrhage	AMAN	Hypertension	IVIG; MV	Partial	1 year	Stand with assistance
Mantero et al., 2013	F/79	Cerebellum hemorrhage	AIDP	Hypertension	IVIG; MV	Partial	6 month	Walk with assistance
Zhang and Li, 2014	M/56	Epidural hematomas	AMAN	Unknown	IVIG	Death	–	–
Wu et al., 2016	F/51	SAH	AMAN	None	IVIG; MV	Partial	1 year	Absent tendon reflexes
	M/68	Hemorrhagic transformation in infarct zone	AIDP	Artery infarction	IVIG; MV	Partial	Unknown	Completely bedridden
Li et al., 2017	F/48	Head injure	AMAN	Unknown	IVIG; MV	Partial	43 days	Muscular atrophy
	F/53	Postoperation of Rathke cyst	AMAN	Unknown	IVIG	Partial	38 days	Muscular atrophy

**SAH, subarachnoid hemorrhage; IVIG, intravenous immunoglobulin; MV, mechanical ventilation; PE, plasma exchange.**

**TABLE 2:** GBS before cerebral hemorrhagic injury.

**References**	**Sex/age**	**Types of CHI**	**Subtype of GBS**	**Vascular dysfunction**	**Treatments**	**Outcome**	**Follow-up time**	**Presentation of prognosis**
Gande et al., 1999	F/47	SAH	AMSAN	Hypertension	IVIG; MV	Partial	2 months	Mobilize with a frame
Doss-Esper et al., 2005	F/66	Basal ganglia hemorrhages and SAH	AMSAN	Hypertension; vasoconstriction in the basilar and bilateral posterior cerebral arteries	IVIG; MV	Partial	3 weeks	Quadriplegic and areflexic
Chang et al., 2014	M/44	Hemorrhagic transformation in infarct zone	MFS	None	IVIG	Partial	Unknown	Unknown
Wei et al., 2015	F/51	SAH	AMSAN	Hypertension; segmental narrowing of the cerebral arteries	IVIG	Completed	7 months	–

**SAH, subarachnoid hemorrhage; IVIG, intravenous immunoglobulin; MV, mechanical ventilation; PE, plasma exchange.**

Previous research has discovered that intraventricular hemorrhage can cause a Toll-like receptor 4 (TLR4)- and NF-κB-dependent inflammatory response (Karimy et al., 2017). In addition, it has been reported that the levels of TLR4 and NF-κB are significantly increased in the CSF of GBS patients (Du et al., 2015). Moreover, it has been demonstrated that TLR4 on antigen-presenting cells is upregulated in the experimental autoimmune neuritis (EAN), an animal model of GBS (Gries et al., 2012). TLR4, MyD88, and NF-κB mRNA expression are also significantly increased in patients with GBS (Wang et al., 2012; Du et al., 2015). MyD88 and NF-κB are two key molecules of the TLR4 signaling pathway. The activated TLR4 signaling pathway can promote the secretion of inflammatory molecules, such as TNF-α, IL-6, IL-8, IL-12, IL-23, and IL-1β (Du et al., 2015; Ebrahim Soltani et al., 2019). Previous researches have shown that IL-1β, TNF-α, and IL-6 are elevated in the EAN rats and GBS patients (Zhang et al., 2007; Hayashi et al., 2008; Matsui et al., 2013; Sun et al., 2019). TNF-α can inhibit Schwann cell proliferation and potentiate Schwann cell apoptosis (Boyle et al., 2005). IL-1β is increased on Schwann cell membranes in GBS patients (Hayashi et al., 2008) and can also enhance the T-cell-dependent response, which further increases the antibody affinity to self-ganglioside (Ebrahim Soltani et al., 2019). IL-6 production is enhanced in the CSF and circulation in acute GBS, which may contribute to acute neuropsychological changes (Ebrahim Soltani et al., 2019). Moreover, other inflammatory cytokines, like interferon-γ, IL-4, IL-17, IL-23, and IL-8, are increased after GBS (Debnath et al., 2018; Breville et al., 2019; Sun et al., 2019). Recent study also reported that the expression of microRNA-146a, which was correlated with IL-6 and TNF-a, was higher in the GBS (Huang et al., 2020). These inflammatory cytokines ultimately induce demyelination, nerve lesions, and axonal degeneration and result in the development of GBS. In our case, the relatives of the patient denied any other previous diseases of the patient, such as hypertension. However, before the patient was discharged from the hospital, head CTA showed cerebral arteriosclerosis. The vascular atherosclerosis in our case may also be associated with a specific inflammatory profile. Thus, the TLR4 and NF-κB signaling pathway may be involved in the pathogenesis of GBS after intraventricular hemorrhage. However, to determine if this pathway is involved in the development of GBS after intraventricular hemorrhage, it will require funding as well as extended patient stays to enable more thorough examinations.

There are some GBS cases that have been described before CHI. The associated mechanisms underlying these cases are worthy to be explored. Vascular dysfunction may be involved in the pathogenesis of CHI after GBS. Previous studies have shown that systemic vascular autonomic function in GBS might be abnormal (Flachenecker et al., 1997; Gande et al., 1999). Vasoconstriction or narrowing of cerebral arteries was presented in GBS cases before CHI (Doss-Esper et al., 2005; Wei et al., 2015). Reversible cerebral vasoconstriction syndrome is also reported in GBS (Riancho et al., 2013; Wei et al., 2015; Shen et al., 2018). The rupture of cerebral microvessels in GBS patients may have occurred due to the impaired vascular autonomic function. Moreover, the adverse effects of immunoglobulin may be associated with the CHI after GBS. Immunoglobulin can cause hemodynamic changes, such as hypotension, hypertension, and tachycardia (Guo et al., 2018; Vitiello et al., 2019). Thromboembolic complications are another adverse effect of immunoglobulin with an incidence of 1–16.9% (Guo et al., 2018). High blood viscosity, hypercoagulopathy, vasospasm, and autoimmune vasculitis are considered as the potential mechanisms. These changes in blood vessel can cause vascular dysfunction as well. Posterior reversible encephalopathy syndrome (PRES) is a rare neurological adverse effect of immunoglobulin (Guo et al., 2018). PRES was also reported in MFS patient with immunoglobulin therapy (Ribeiro et al., 2016). The dilation and constriction in the secondary and tertiary branches of intracranial artery is one of neuroimaging features of PRES (Chen et al., 2016). Thus, vascular dysfunction caused by GBS and immunoglobulin may be involved in the pathogenesis of cerebral hemorrhagic injury after GBS. However, further researches are still needed.

The differential diagnosis in our case is also an issue worthy of discussion. Based on the symptoms, the diagnosis of critical illness polyneuropathy (CIP) or critical illness myopathy (CIM) was also considered. CIP and CIM are complications of a severe critical illness and cause a failure to wean from the ventilator in the ICU. CIP is a distal axonal sensory-motor polyneuropathy that presents with weakness of the bilateral limbs and respiratory muscles (Latronico and Bolton, 2011; Murayama et al., 2019). It is usually associated with sepsis, systemic inflammatory response syndrome, and/or multi-organ failure. The electrophysiological features of CIP are decreased amplitudes of compound muscle action potentials and nerve conduction velocities of sensory nerve action potentials (Latronico and Bolton, 2011). CIM is a primary myopathy with similar clinical features of CIP, but without the sensory involvement. An increased duration of compound muscle action potential amplitudes, normal sensory nerve action potentials, and reduced muscle excitability are the major electrophysiological features of CIM (Latronico and Bolton, 2011). A muscle biopsy may also be helpful for diagnosis of CIP/CIM, but albuminocytologic dissociation is usually not present in the CSF of CIP/CIM. However, hemorrhage and neurosurgery may influence the results from the CSF. Therefore, CIM/CIP could not be completely ruled out without pathological evidence. A creatinine kinase test may also be helpful for the distinction of CIM/CIP from GBS. In addition, hypokalemia was found in our case and, when combined with the low potassium levels, made the paralysis another potential diagnosis. Hypokalemic periodic paralysis is a neuromuscular disease characterized by episodic attacks of flaccid weakness (Statland et al., 2018). The clinical features of hypokalemic periodic paralysis can last hours to days; however, they are periodic (Statland et al., 2018). The pathogenic mechanism of hypokalemic periodic paralysis is primarily concerned with gene mutations and the diagnosis can be confirmed by testing for specific genes. In our case, the hypokalemia had been corrected, but the flaccid paralysis persisted. Other diseases, such as myasthenia gravis and paraneoplastic neurological syndrome, should not be ignored. Although multiple etiologies account for limb and respiratory muscle weakness in the ICU, GBS after spontaneous intraventricular hemorrhage is rare. However, as a life-threatening complication of intraventricular hemorrhage, GBS should receive more attention.

The prognosis of most patients with GBS is good, but mortality rates are still between 4 and 5%. Ventilatory insufficiency, pulmonary complications, and autonomic dysfunction are among the most common complications of GBS that can result in death (Willison et al., 2016). The quality of patients’ lives suffers dramatically, which manifest as disability, reduced myodynamia, pain, and fatigue (Willison et al., 2016). Moreover, the prognosis of GBS combined with cerebral hemorrhagic injury is even worse. Nearly 90% of the cases in the literature show only partial recovery or even death (Table 1). In our case, intraventricular hemorrhage and mechanical ventilation may be the major factors leading to the poor prognosis. Even though the overall mortality rate is relatively low, GBS is still a life-threatening disorder, especially after cerebral hemorrhagic injury, and requires further research to enable the development of therapeutics for GBS.

## Limitations

The electromyogram examination was absent because of substantial logistical difficulties at that time. This may influence the level of diagnostic certainty. Moreover, the electromyogram examination is also helpful for the exclusion of several other diseases. The albuminocytologic dissociation was a para-clinic criterion for the diagnostic in this case. However, the results from the CSF may be explained by hemorrhage and neurosurgery. The cause of albuminocytologic dissociation needs depth analyses.

## Conclusion

GBS after spontaneous intraventricular hemorrhage is a rare occurrence. The pathogenic mechanism of GBS has been discussed for more than a century and remains undiscovered. The TLR4 and NF-κB signaling pathway may be involved in the pathogenesis of GBS after intraventricular hemorrhage, but additional research and information is required to prove this pathway’s involvement in the development of GBS after intraventricular hemorrhage. In the ICU, a number of diseases present with limb and respiratory muscle weakness. However, as a complication of cerebral hemorrhagic injury, GBS requires more attention for the differential diagnosis of limb and respiratory weakness. Although the prognosis of most patients with GBS is usually good, cerebral hemorrhage and mechanical ventilation may be risk factors that exacerbate the condition.

## Data Availability Statement

All datasets generated for this study are included in the article.

## Ethics Statement

The studies involving human participants were reviewed and approved by The Second Affiliated Hospital, School of Medicine, Zhejiang University. The patients/participants provided their written informed consent to participate in this study. Written informed consent was obtained from the individual(s) for the publication of any potentially identifiable images or data included in this article.

## Author Contributions

JH, XL, and YW collected the data and drafted the manuscript. EP, AS, and QF reviewed and modified the manuscript. All authors contributed to the article and approved the submitted version.

## Conflict of Interest

The authors declare that the research was conducted in the absence of any commercial or financial relationships that could be construed as a potential conflict of interest.

## Abbreviations

AIDP: acute inflammatory demyelinating polyneuropathy
AMAN: acute motor axonal neuropathy
AMSAN: acute motor-sensory axonal neuropathy
CHI: cerebral hemorrhagic injury
CIM: critical illness myopathy
CIP: critical illness polyneuropathy
CSF: cerebrospinal fluid
CT: computer tomography
CTA: computed tomography angiography
GBS: Guillain–Barré syndrome
ICU: intensive care unit
MFS: Miller Fisher syndrome
TLR4: Toll-like receptor 4.
